# Digital Ischemia as a Rare Initial Presentation of Myeloperoxidase (MPO)-Anti-neutrophil Cytoplasmic Antibody (ANCA)-Positive Granulomatosis With Polyangiitis: A Case Report

**DOI:** 10.7759/cureus.107593

**Published:** 2026-04-23

**Authors:** Ryan K Penaflor, Johnson Ong, Mindy Phan, Sana Afroz

**Affiliations:** 1 Internal Medicine, Riverside University Health Systems, Moreno Valley, USA; 2 Rheumatology, Loma Linda University Health, Loma Linda, USA

**Keywords:** atypical gpa, digital ischemia, granulomatosis with polyangiitis (gpa), granulomatous vasculitis, mpo-anca/p-anca-associated vasculitis

## Abstract

Anti-neutrophil cytoplasmic antibody (ANCA)-associated vasculitides (AAV), particularly myeloperoxidase (MPO)-ANCA-positive granulomatosis with polyangiitis (GPA), rarely present with acute arterial digital ischemia or necrosis.* *

We report a 68-year-old woman with a history of seropositive rheumatoid arthritis, suspected connective tissue disease (CTD)-associated interstitial lung disease, scleromalacia perforans, and osteoporosis who presented with acute ischemia of the left hallux that progressed to involve multiple digits despite therapeutic anticoagulation. Evaluation for cardioembolic, malignant, infectious, and hypercoagulable etiologies was unrevealing.Serologic testing demonstrated elevated rheumatoid factor (660 IU/mL), positive P-ANCA with elevated MPO antibodies (55.2 U), erythrocyte sedimentation rate of 130 mm/hr, C-reactive protein of 5.25 mg/L, and negative proteinase-3 (PR3) antibodies. A skin biopsy of the affected left hallux showed small- and medium-vessel wall inflammation with lymphoplasmacytic and granulomatous infiltrates consistent with GPA.

The patient was treated with pulse-dose intravenous methylprednisolone followed by high-dose oral prednisone, which halted the progression of ischemia. Despite this, she required amputation of the left hallux due to dry gangrene after irreversible tissue injury developed. There was no recurrence of ischemia or other systemic vasculitic manifestations after the initiation of rituximab maintenance therapy, with subsequent clinical and serologic remission.

This case highlights isolated arterial digital ischemia as a rare but severe manifestation of MPO-ANCA-positive GPA and underscores the importance of considering vasculitis in patients who present with unexplained digital ischemia, particularly when symptoms progress despite anticoagulation, as early immunosuppressive therapy may prevent further vascular injury.

## Introduction

Anti-neutrophil cytoplasmic antibody (ANCA)-associated vasculitides (AAV) are a group of systemic autoimmune small-vessel vasculitides that include granulomatosis with polyangiitis (GPA), microscopic polyangiitis (MPA), and eosinophilic granulomatosis with polyangiitis (EGPA) [1-7]. These conditions are characterized by circulating autoantibodies directed against neutrophil antigens, most commonly proteinase-3 (PR3) and myeloperoxidase (MPO), and are associated with significant morbidity and mortality if not promptly recognized and treated [1-7]. More broadly, vasculitis refers to inflammation of blood vessel walls that can lead to luminal compromise and tissue ischemia, with distinct phenotypes based on the size of vessels involved.

GPA is a necrotizing granulomatous vasculitis most commonly associated with PR3-ANCA and typically presents with sinonasal involvement, glomerulonephritis, and pulmonary manifestations, including nodules, masses, and cavitary lesions [1,2]. Necrotizing granulomatous inflammation in GPA is characterized by poorly formed granulomas composed of epithelioid histiocytes and multinucleated giant cells with central necrosis, accompanied by necrotizing small- to medium-vessel vasculitis with fibrinoid necrosis and leukocytoclasia, resulting in vessel compromise. Although PR3-ANCA is strongly associated with GPA, an MPO-ANCA-positive phenotype has been described, which is often associated with more limited disease, a higher prevalence of subglottic stenosis, and a female predominance [7]. This MPO-ANCA-positive phenotype may represent a distinct clinical subset with differing clinical characteristics compared to classic PR3-ANCA-positive disease.

Despite these well-described clinical features, acute arterial digital ischemia remains a rare and underrecognized manifestation of GPA. Prior reports have demonstrated that AAV may involve not only the lungs and kidneys but also the venous and arterial vasculature, with thrombotic complications observed in both settings [4,5]. In addition to classic small-vessel inflammation, AAV is increasingly recognized as a prothrombotic inflammatory state [6]. The pathogenesis of thrombosis in GPA, often referred to as immunothrombosis, is not fully understood. Proposed mechanisms include neutrophil activation by circulating anti-PR3 or anti-MPO antibodies. When neutrophils are activated, they adhere to endothelial cells and release reactive oxygen species and proteolytic enzymes, resulting in endothelial injury [6-10]. In addition, activated neutrophils release neutrophil extracellular traps (NETs), which consist of DNA, histones, and granular proteins that form web-like structures and promote thrombogenesis [7-11]. NETs have been shown to enhance tissue factor expression, inhibit anticoagulation pathways, and promote platelet adhesion, thereby amplifying the coagulation cascade and contributing to intravascular thrombosis [7-11]. Endothelial injury may further increase von Willebrand factor release, augmenting platelet activation. Together, these processes provide a plausible mechanism for arterial thrombosis and digital ischemia in patients with active GPA. Given the inflammatory mechanisms underlying vascular occlusion in AAV, treatment typically focuses on immunosuppressive therapy, while antithrombotic therapies may be considered based on individual thrombotic risk [6,11]. 

Recognition of atypical vascular presentations is critical, as delayed diagnosis may result in irreversible tissue injury. Here, we report a case of MPO-ANCA-positive GPA presenting with acute digital arterial ischemia leading to toe necrosis, highlighting a rare but clinically significant manifestation and emphasizing the importance of early recognition when ischemia progresses despite anticoagulation.

## Case presentation

A 68-year-old woman with a history of seropositive rheumatoid arthritis complicated by scleromalacia perforans, suspected connective tissue disease-associated interstitial lung disease (CTD-ILD) on home oxygen, and osteoporosis with vertebral compression fractures was transferred from an outside hospital for the evaluation of acute left hallux ischemia. 

The patient reported sudden-onset left hallux pain four days prior to presentation, associated with coldness of the digit. Two days later, symptoms progressed with worsening pain and the development of dusky discoloration, prompting medical evaluation. She denied trauma, similar prior episodes, or new systemic symptoms. She reported baseline exertional dyspnea requiring 3 L supplemental oxygen without acute respiratory changes.

**Figure 1:**
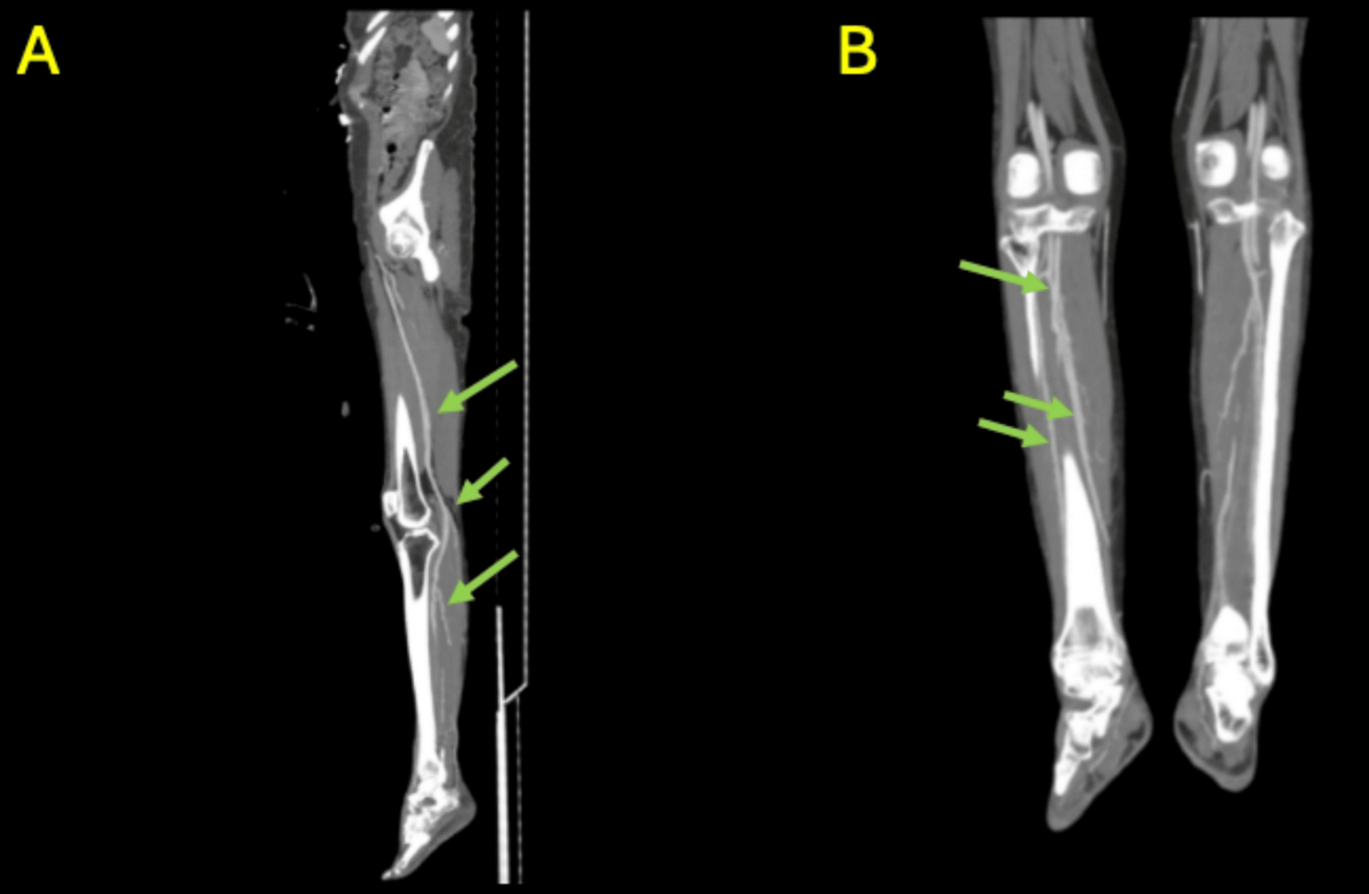
Computed tomography angiography of bilateral lower extremities In both sagittal view (A) and coronal view (B), there is preserved arterial flow bilaterally without large-vessel occlusion (denoted by green arrows).

Initial workup at the outside hospital showed computed tomography (CT) angiography of the lower extremities, which demonstrated preserved arterial flow without significant occlusion or stenosis (Figure 1A-1B). 

**Figure 2:**
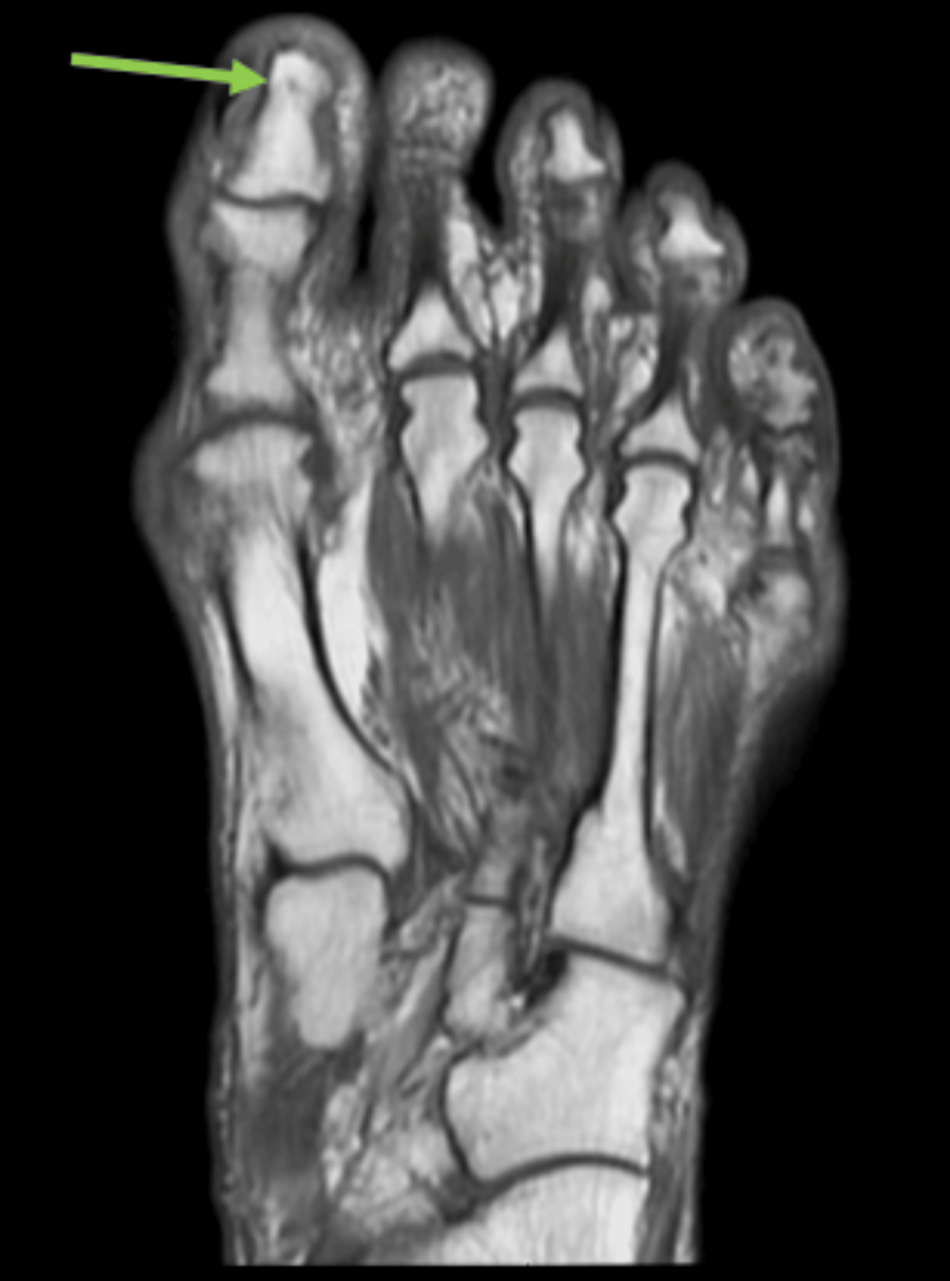
Magnetic resonance imaging of the left foot (with and without contrast) There is no evidence of osteomyelitis, abscess, fracture, or soft tissue infection (denoted by green arrow).

Physical examination revealed palpable dorsalis pedis and posterior tibial pulses bilaterally, but was notable for a cold, cyanotic left hallux. Magnetic resonance imaging (MRI) of the left foot showed no evidence of osteomyelitis, abscess, or fracture (Figure 2).

**Figure 3:**
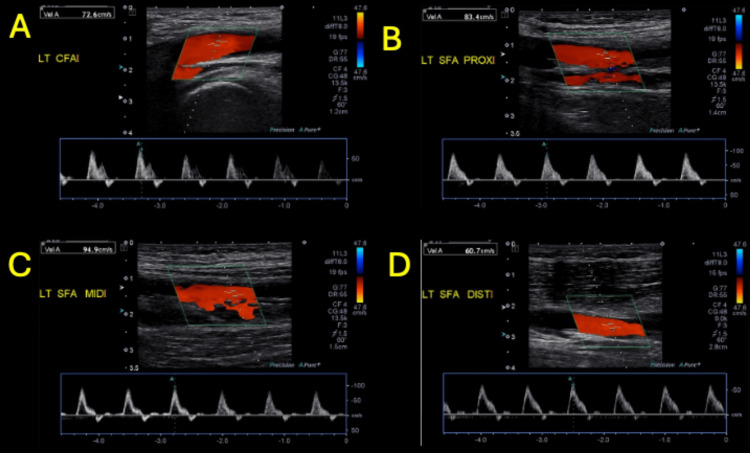
Left lower extremity arterial duplex ultrasound (part 1) Left common femoral artery (LT CFA) with triphasic waveform and peak systolic velocity 67 cm/s (A). Left superficial femoral artery proximal, middle, and distal (LT SFA PROX, MID, DIST) with triphasic waveform and peak systolic velocity 135 cm/s (B, C, D).

**Figure 4:**
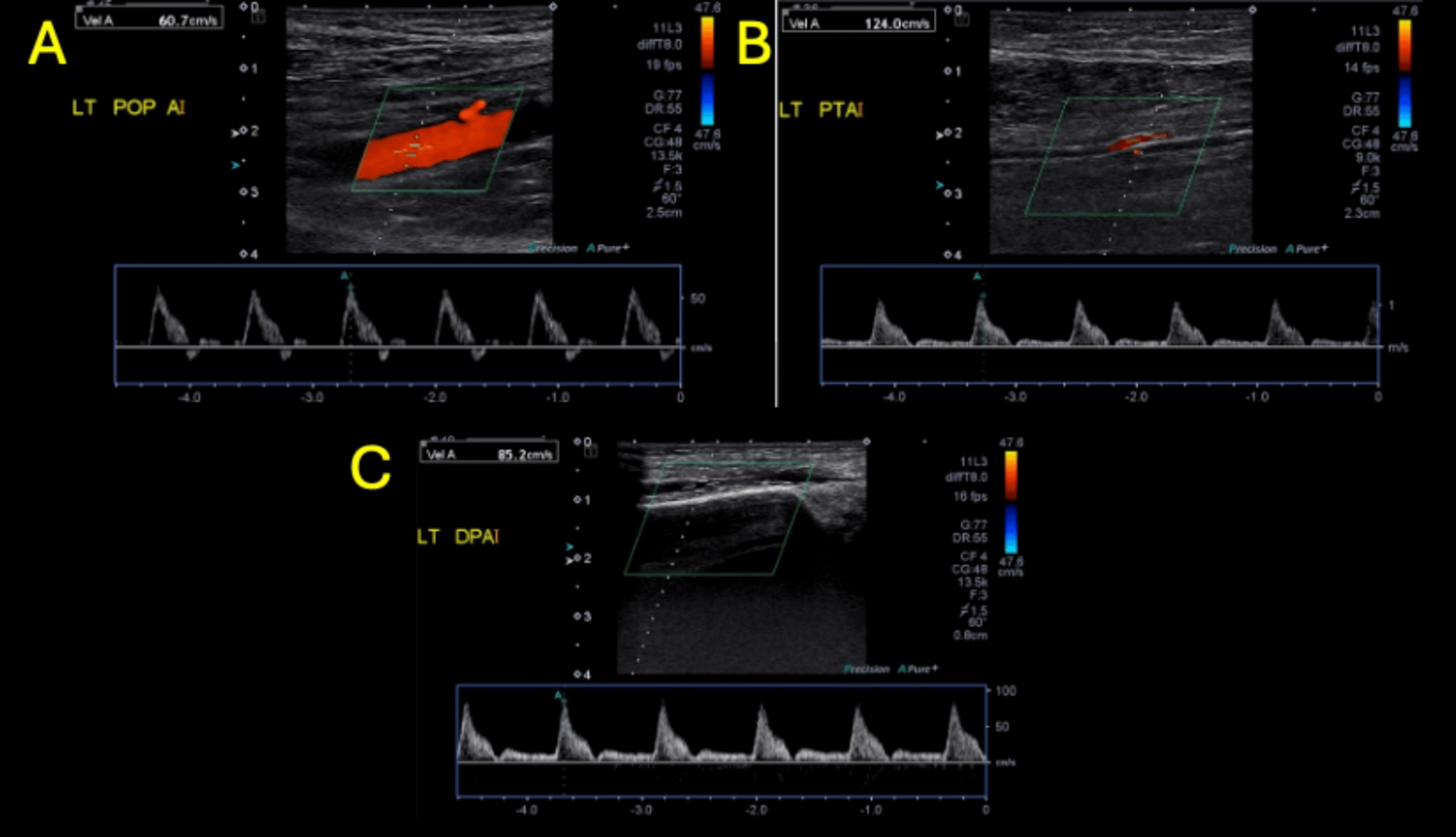
Left lower extremity arterial duplex ultrasound (part 2) Left popliteal artery (LT POP A) with triphasic waveform and peak systolic velocity 74 cm/s (A). Left posterior tibial artery (LT PTA) with monophasic waveform 105 cm/s (B). Left dorsalis pedis artery (LT DPA) with monophasic waveform and peak systolic velocity 120 cm/s (C).

Lower extremity arterial duplex ultrasound showed mild distal peripheral arterial disease with monophasic waveforms in the left lower extremity; otherwise, there was no high-grade focal stenosis or occlusion (Figure 3A-3D and Figure 4A-4C). 

**Figure 5:**
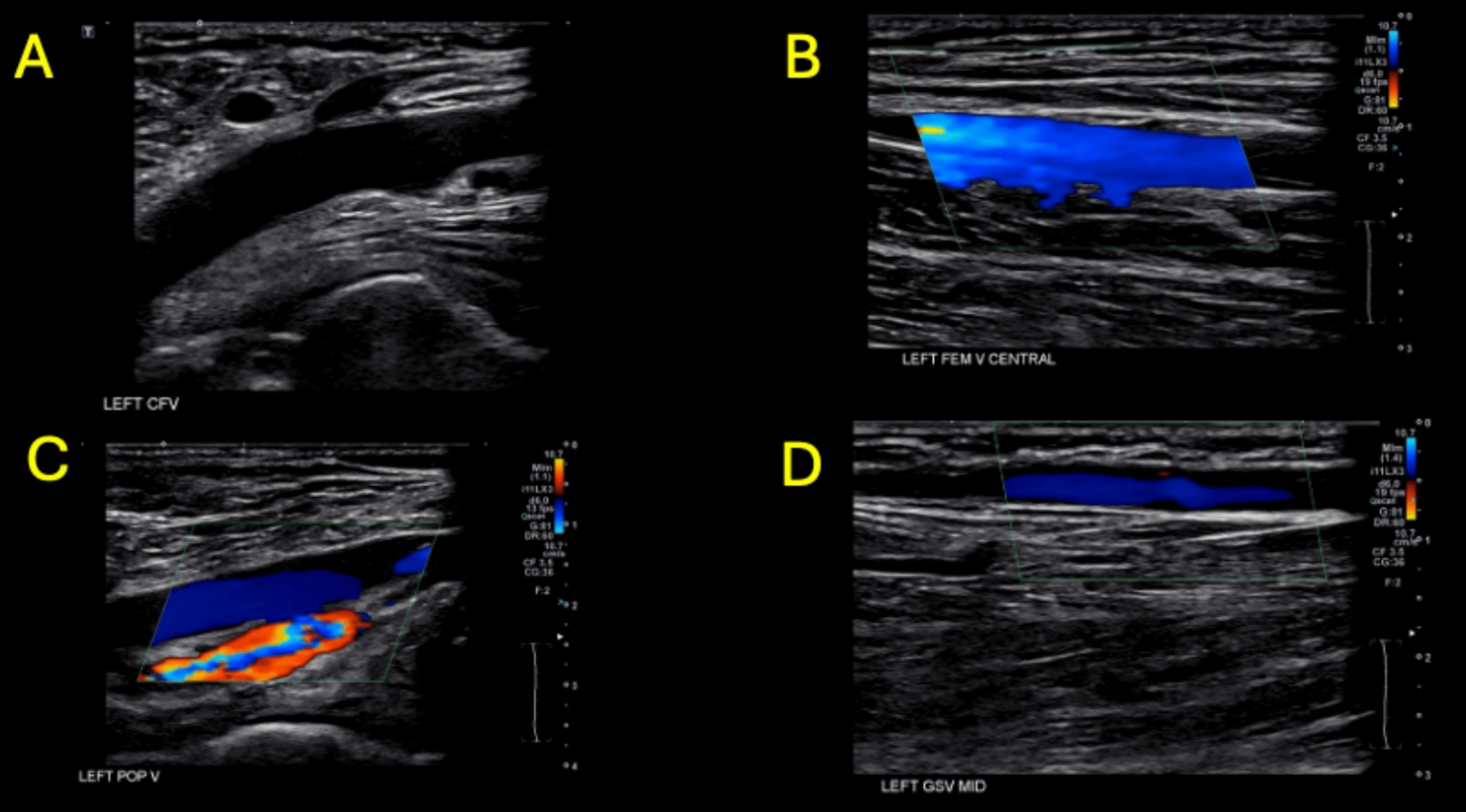
Left lower extremity venous duplex ultrasound There is no evidence of deep vein thrombosis or venous reflux within the left common femoral vein (CFV) (A), left femoral vein central (FEM V CENTRAL) (B), left popliteal vein (POP V) (C), and left middle great saphenous vein (GSV MID) (D).

Lower extremity venous duplex ultrasound showed no evidence of deep vein thrombosis or venous reflux bilaterally (Figure 5A-5D).

**Figure 6:**
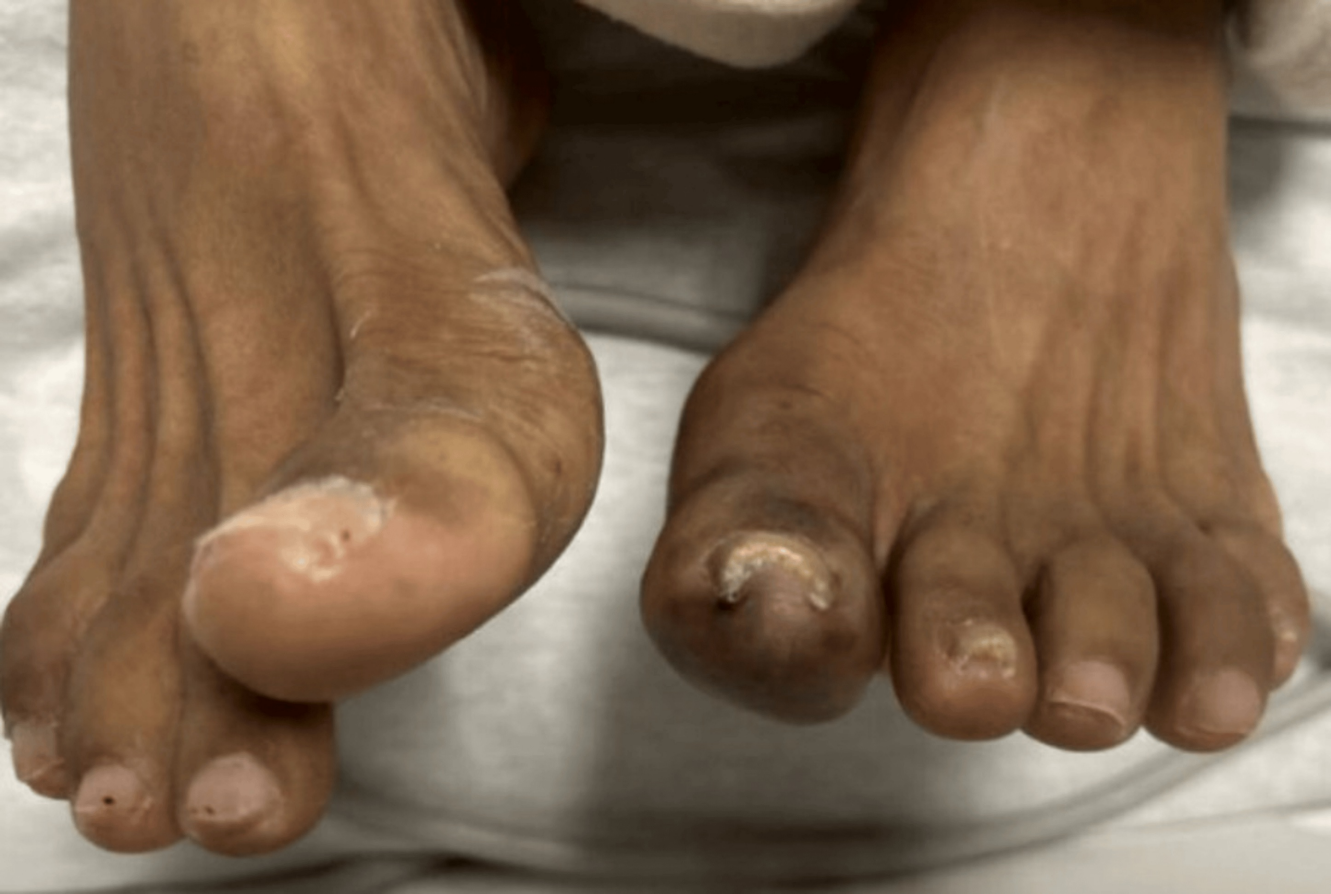
Day 1: left hallux ischemia on presentation

**Figure 7:**
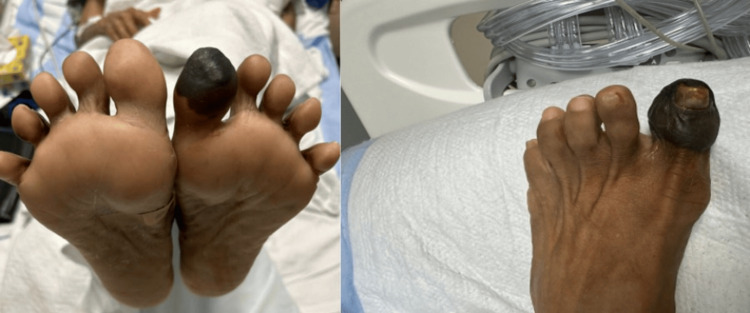
Day 12: progression and worsening of left hallux ischemia despite anticoagulation

**Figure 8:**
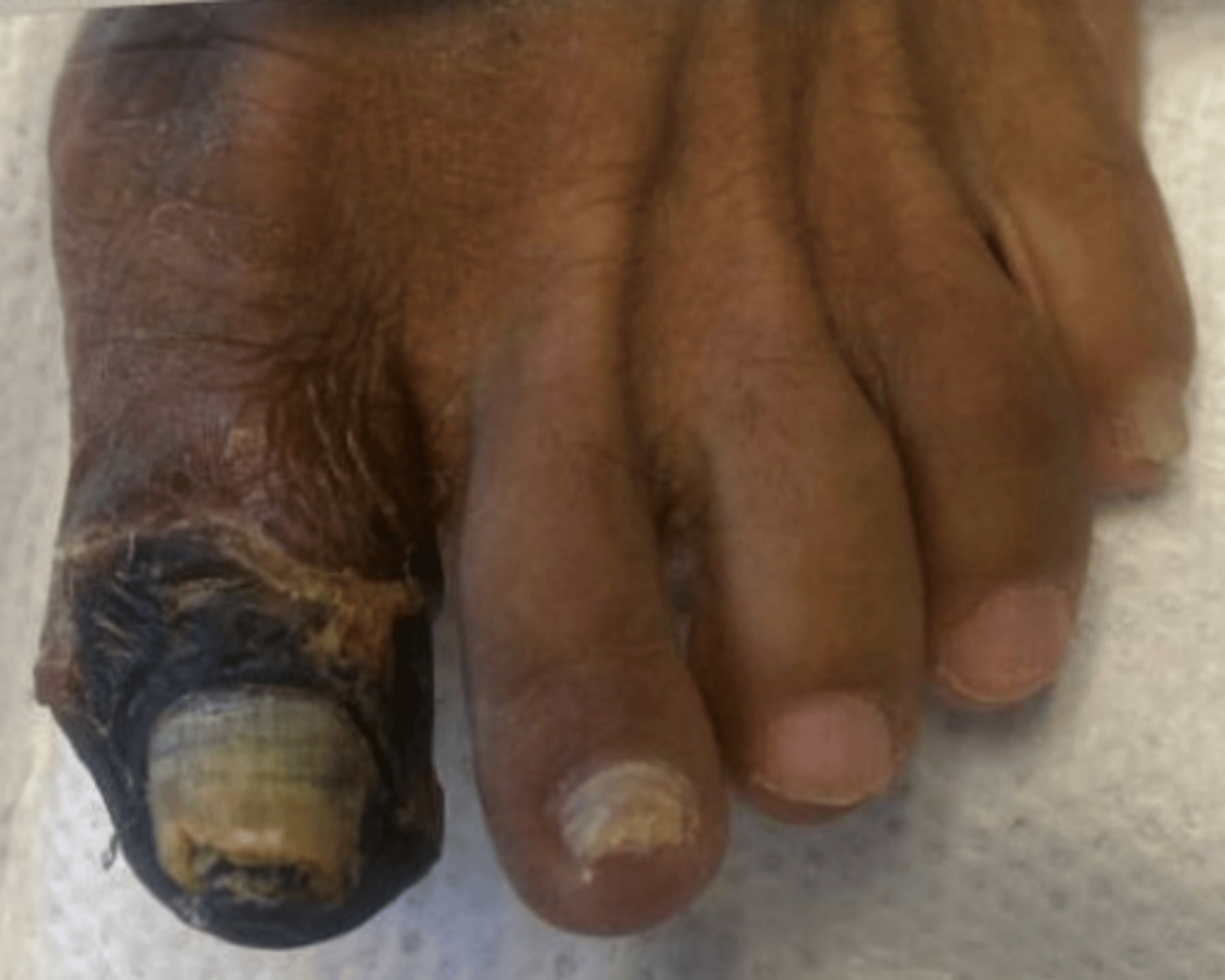
Day 45: dry gangrene and necrosis post-hospitalization following the initiation of immunosuppressive therapy

A heparin infusion was initiated due to concern for embolic or thrombotic ischemia. Despite therapeutic anticoagulation, her condition progressed. By hospital day 12, she developed new ischemic changes in multiple digits of the right foot, along with worsening ischemia of the left hallux (Figures 6-8). The progression of ischemia despite therapeutic anticoagulation raised concern for an inflammatory or vasculitic etiology rather than a primary thromboembolic process.

Renal function remained normal throughout hospitalization. Serologies revealed elevated rheumatoid factor of 660 IU/mL, positive P-ANCA (1:40), elevated MPO antibody (55.2 U), and negative PR3 antibody. Inflammatory markers were elevated, with an erythrocyte sedimentation rate of 130 mm/hr and C-reactive protein of 5.25 mg/L, consistent with systemic inflammation (Table 1).

**Table 1:** Relevant laboratory and serologic findings Summary of laboratory and serologic findings relevant to the evaluation of arterial digital ischemia. Of note, protein C/protein S/antithrombin III levels may be affected by acute illness and anticoagulation. ESR: erythrocyte sedimentation rate; CRP: C-reactive protein; MPO: myeloperoxidase; PR3: proteinase-3; anti-CCP: anti-cyclic citrullinated peptide; P-ANCA: perinuclear anti-neutrophil cytoplasmic antibodies; IFA: immunofluorescence assay

Test	Result	Reference range
Rheumatoid factor	660 IU/mL	<20 IU/mL
Anti-CCP antibody	Negative (<16 IU/mL)	<16 IU/mL
ESR	130 mm/hr	0-30 mm/hr
CRP	5.25 mg/L	<5 mg/L
P-ANCA (IFA)	Positive (1:40)	<1:20 titer
MPO antibody	55.2 U	<20 U
PR3 antibody	Negative	Negative
Anticardiolipin antibody	Negative	Negative
β2-Glycoprotein I antibody	Negative	Negative
Lupus anticoagulant	Negative	Negative
Complement C3	Normal	90-180 mg/dL
Complement C4	Normal	10-40 mg/dL
Protein S	64%	65-130%
Protein C	37%	70-180%
Antithrombin III	75%	80-140%
Cryoglobulins	Negative	Negative
Urinalysis	Negative	Negative
Urine protein, random	7.6	<20 mg/dL
Urine creatinine, random	26	30-300 mg/dL
Urine protein/creatinine ratio	292 mg/g (≈0.3 g/day)	<200 mg/g, <0.2 g/day
Hepatitis B	Core IgM positive, surface antibody positive, surface antigen negative (consistent with prior resolved infection)	Negative
Hepatitis C	Negative	Negative
HIV-1/2 Ag/Ab (fourth generation)	Negative	Negative

**Figure 9:**
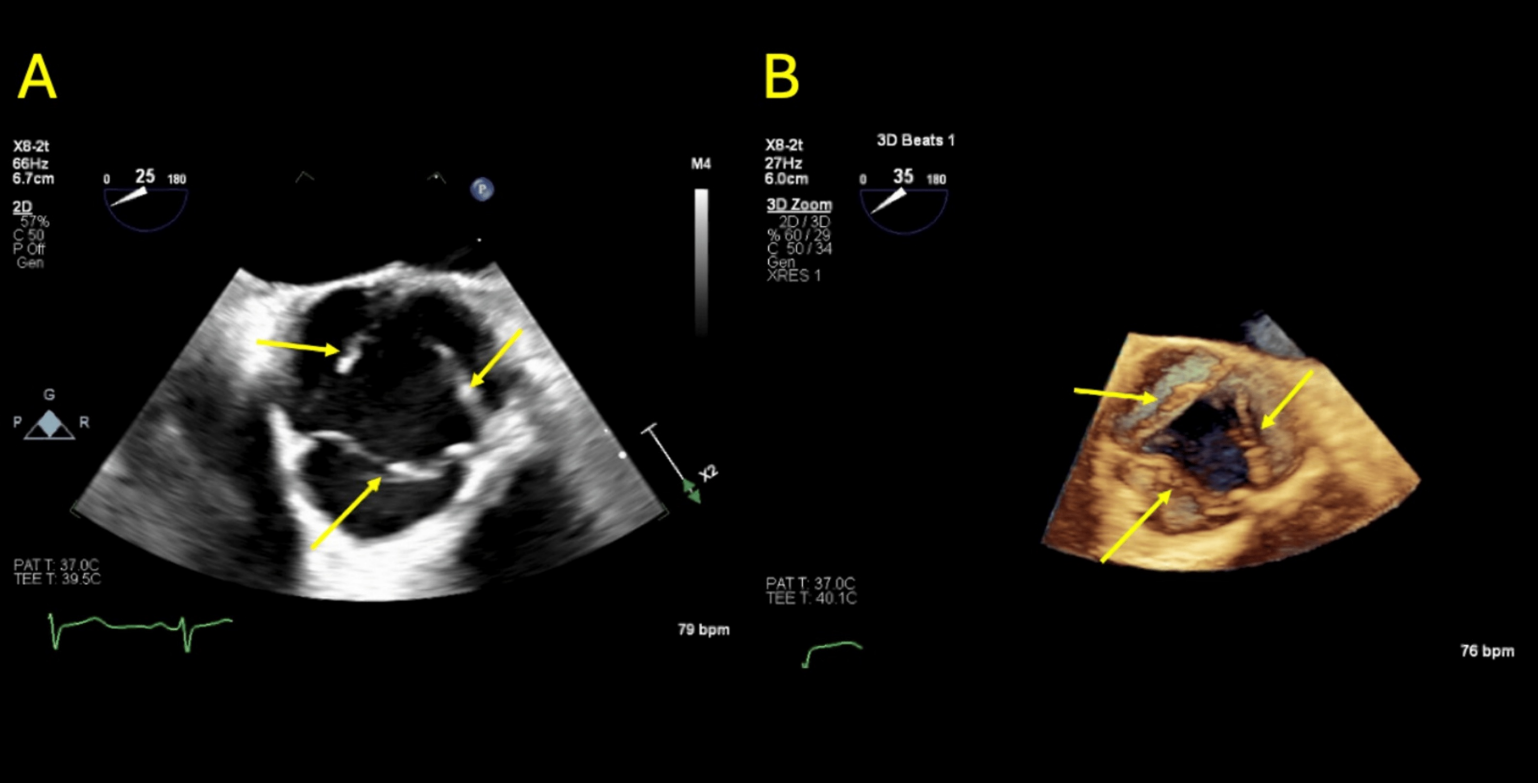
Mid-esophageal transesophageal echocardiogram short-axis view of the aortic valve On both 2D echocardiographic view (A) and 3D echocardiographic view (B), there are multiple small, nodular, sessile excrescences along the lines of coaptation, likely nonbacterial thrombotic (marantic) vegetations (denoted by yellow arrows).

Furthermore, workup for embolic and hypercoagulable etiologies was unrevealing. Transthoracic echocardiogram (TTE) showed no evidence of intracardiac thrombus or valvular vegetations. Transesophageal echocardiogram (TEE) identified small nonbacterial thrombotic (marantic) vegetations, which were deemed unlikely to account for the patient's digital ischemia per cardiology consultation (Figure 9A-9B). 

**Figure 10:**
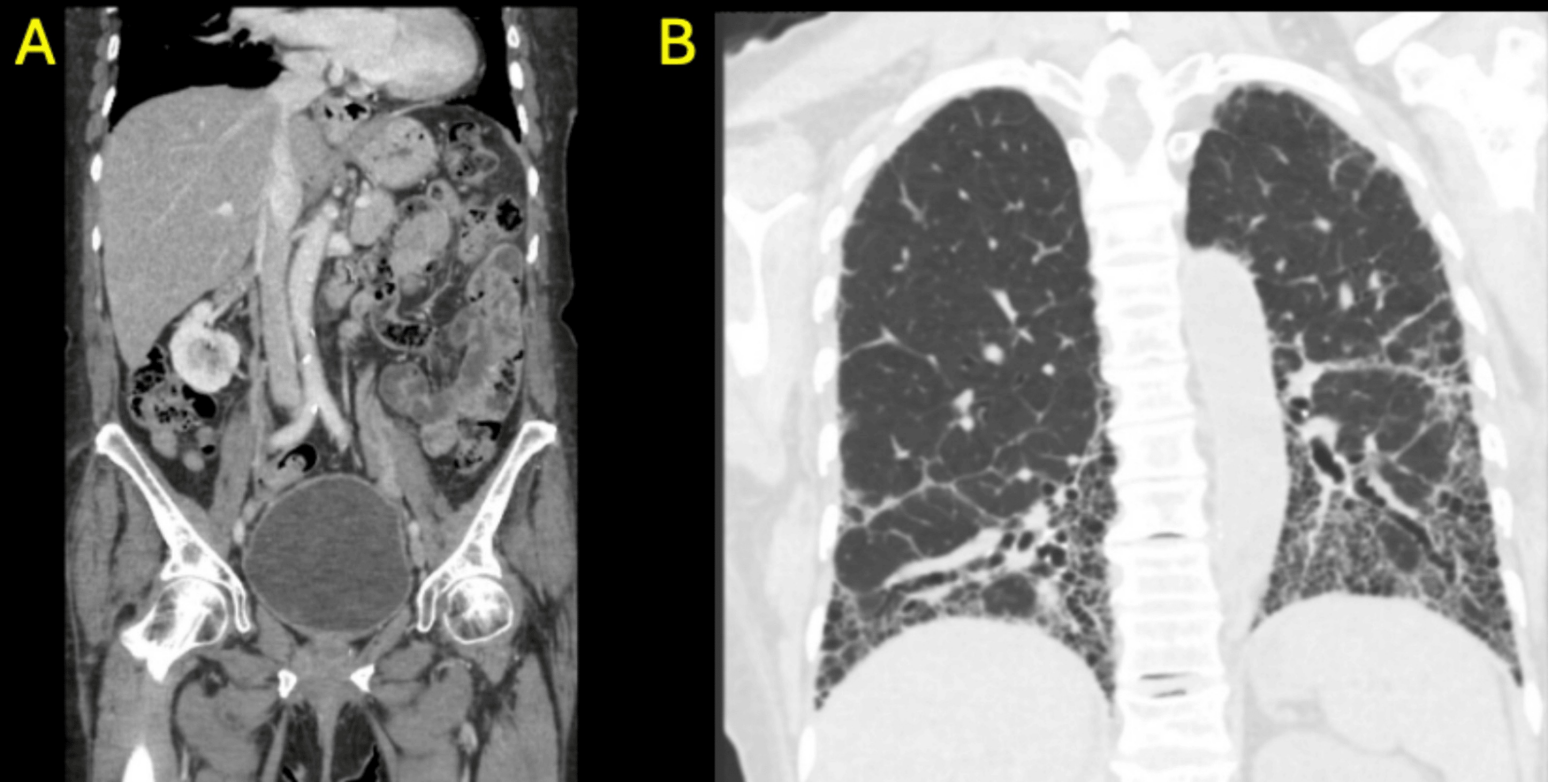
CT of the abdomen/pelvis with contrast and CT of the chest without contrast CT of the abdomen/pelvis with contrast (A) did not demonstrate malignancy but showed multilevel thoracolumbar spondylosis and diffuse hepatic steatosis. CT of the chest without contrast (B) showed extensive subpleural fibrosis of lower lobes left-greater-than-right with extensive honeycombing and bronchiectatic changes in lower lobes, right middle lobe, and left upper lobe. CT: computed tomography

Hypercoagulable evaluation, including antiphospholipid antibody testing, was negative, and malignancy screening with CT of the chest, abdomen, and pelvis was unremarkable (Figure 10A-10B). 

**Figure 11:**
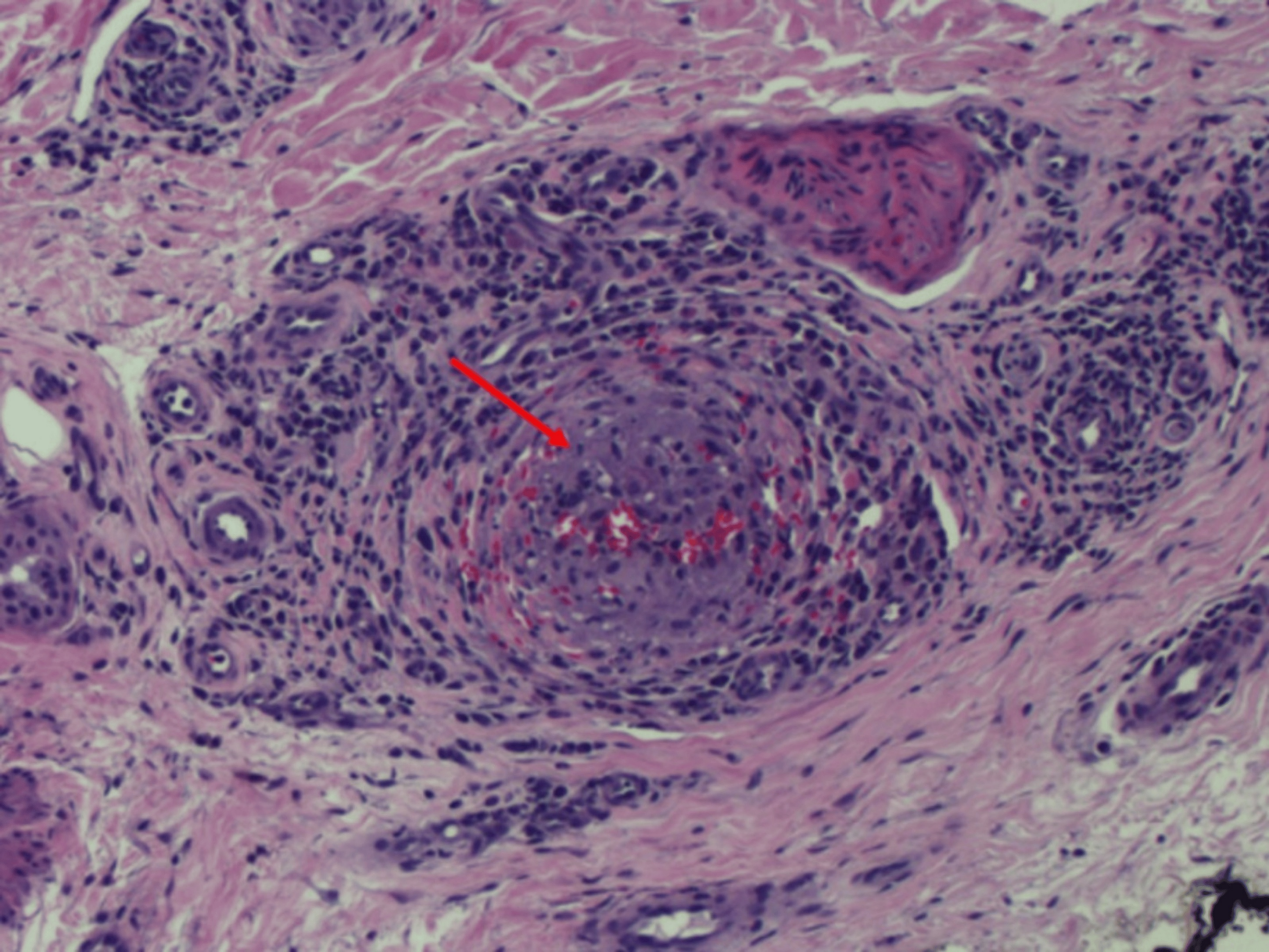
Left hallux biopsy H&E stain (100×) Medium-sized dermal vessel with granulomatous inflammation. An epithelioid granuloma nearly occludes the vessel lumen (denoted by red arrow), with surrounding lymphoplasmacytic inflammation. There is chronic inflammation, predominantly lymphocytoplasmacytic, within the vessel wall and around the vessel.

Given progression despite anticoagulation and absence of an embolic source, a vasculitic etiology was suspected. The patient was treated with intravenous methylprednisolone (500 mg daily for three days), followed by oral prednisone (1 mg/kg daily). A skin biopsy of the left hallux demonstrated small- and medium-vessel wall inflammation with lymphoplasmacytic and granulomatous infiltrates consistent with GPA (Figure 11).

**Figure 12:**
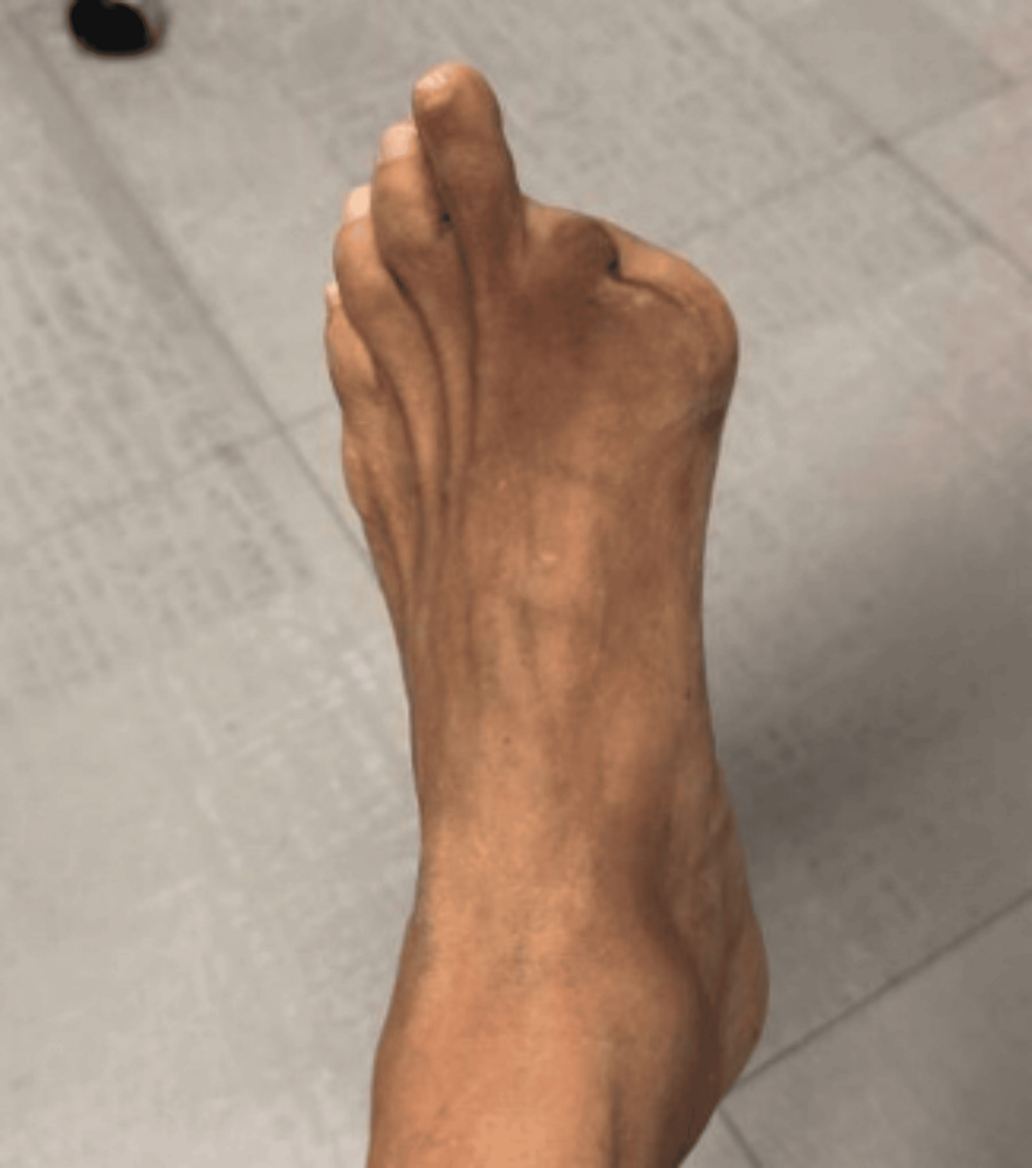
One-year follow-up demonstrating status post transmetatarsal amputation of the left hallux

Following the initiation of corticosteroid therapy, the progression of ischemia halted promptly, and no new areas of involvement were observed. However, she subsequently developed dry gangrene of the left hallux, requiring transmetatarsal amputation months later (Figure 12). On the outpatient follow-up, the patient was initially managed with prednisone taper and mycophenolate mofetil by an outside rheumatologist. She later re-established care at our institution, where maintenance intravenous rituximab was initiated with two 500 mg infusions separated by two weeks and planned intravenous rituximab 500 mg every six months. Hematology evaluation did not support a diagnosis of antiphospholipid syndrome, and anticoagulation was discontinued in favor of low-dose aspirin.

At the most recent follow-up, the patient has had no recurrence of digital ischemia or other systemic vasculitic manifestations. Repeat ANCA testing was negative, consistent with serologic remission. She continues to follow up with rheumatology for the management of MPO-ANCA-positive GPA with an atypical presentation of digital ischemia (Table 2).

**Table 2:** Key clinical findings, diagnostic workup, and differential diagnosis in MPO-ANCA-positive GPA presenting as digital ischemia PAD: peripheral arterial disease; CT: computed tomography; PTA: posterior tibial artery; DPA: dorsalis pedis artery; DVT: deep vein thrombosis; TTE: transthoracic echocardiography; TEE: transesophageal echocardiography; C/A/P: chest, abdomen, and pelvis; ESR: erythrocyte sedimentation rate; CRP: C-reactive protein; MPO: myeloperoxidase; ANCA: anti-neutrophil cytoplasmic antibody; PR3: proteinase-3; RF: rheumatoid factor; GPA: granulomatosis with polyangiitis; MPA: microscopic polyangiitis; RA: rheumatoid arthritis; P-ANCA: perinuclear anti-neutrophil cytoplasmic antibodies

Domain	Key findings	Clinical significance	Major differential diagnoses considered
Symptoms on presentation	Acute left hallux pain, coldness, and cyanosis	Progressive arterial insufficiency refractory to anticoagulation, raising concern for vasculitis or microvascular thrombosis	Embolic disease, atherosclerotic PAD, hypercoagulable state from malignancy, traumatic injury, vasculitis
Large-vessel imaging	CT angiography of lower extremities was negative without occlusion or stenosis	Ruled out large-vessel thromboembolism or occlusive PAD	Embolic arterial occlusion, acute and/or chronic limb ischemia from PAD, atherosclerotic disease, small-vessel vasculopathy, small-vessel vasculitis, thromboangiitis obliterans, DVT-related phlegmasia, venous outflow obstruction
Peripheral arterial studies	Left lower extremity arterial duplex ultrasound with monophasic distal waveforms (PTA, DPA)	Suggestive of distal microvascular compromise rather than proximal occlusion.	
Venous imaging	Left lower extremity venous duplex ultrasound negative for DVT or venous reflux	Ruled out DVT or venous congestion as cause of ischemia	
Cardiac evaluation	TTE/TEE negative for intracardiac thrombus with the presence of small nonbacterial (marantic) vegetations	Low likelihood of cardioembolic source; marantic vegetations were deemed clinically insignificant	Infective endocarditis, cardioembolic disease, nonbacterial thrombotic endocarditis (Libman-Sacks), atrial thrombus
Malignancy screening	CT C/A/P negative for malignancy	Reduced likelihood of paraneoplastic thrombosis or thromboembolism associated with malignancy	Malignancy-associated hypercoagulability
Inflammatory markers	ESR 130 mm/hr, CRP mildly elevated (5.25 mg/L)	Marked systemic inflammatory signal suggestive of an autoimmune or vasculitic process	Infection, autoimmune flare, malignancy
Autoimmune serology	MPO-ANCA positive (55.2 U), P-ANCA positive; PR3 negative; RF markedly elevated	Supported MPO-ANCA-associated vasculitis phenotype of GPA; RF elevation confounds RA overlap	GPA (MPO-variant vs. PR3-variant), MPA, rheumatoid vasculitis
Hypercoagulable workup	Antiphospholipid antibodies negative; low protein C/protein S/antithrombin III (likely acquired due to acute inflammation)	Argued against primary thrombophilia	Antiphospholipid syndrome, inherited thrombophilia
Renal status	Renal function normal and intact without escalating proteinuria or hematuria	Suggestive of limited GPA phenotype without glomerulonephritis	MPA, systemic GPA, renal vasculitis, nephritic or nephrotic syndrome
Skin biopsy (hallux)	Small- and medium-vessel vasculitis with granulomatous inflammation and vessel occlusion	Diagnostic of necrotizing granulomatous vasculitis consistent with GPA	Small-vessel vasculitis due to GPA vs. MPA (less likely due to granulomas), rheumatoid vasculitis
Response to therapy	Progression of ischemia halted with high-dose steroids and no response to anticoagulation	Strong evidence of immunothrombosis-related vascular injury rather than thromboembolism	Thromboembolic disease (ruled out), vasospastic disease, small-vessel vasculitis due to GPA vs. MPA
Synthesis of findings	Progressive ischemia without embolic source, positive MPO-ANCA serologies, granulomatous findings on pathology	Findings consistent with MPO-ANCA-positive GPA presenting as isolated digital ischemia due to immunothrombotic small- to medium-vessel occlusion	

## Discussion

Digital ischemia progressing to necrosis and ultimately gangrene is a rare manifestation of MPO-ANCA-positive GPA and is more commonly associated with large- or medium-vessel vasculitides, embolic disease, or advanced atherosclerosis [10-12]. Although increasing literature has described arterial thrombosis in AAV in the absence of identifiable embolic or hypercoagulable etiologies, this presentation remains uncommon, particularly in MPO-ANCA-positive GPA.

In this case, extensive multidisciplinary investigation failed to identify a cardioembolic source, malignancy, infection, or antiphospholipid syndrome. Notably, the patient had preserved distal pulses and unremarkable large-vessel imaging. Despite therapeutic anticoagulation, ischemia progressed to involve multiple digits in both feet. This lack of response to anticoagulation, combined with supportive serologic and histopathologic findings, favored a vasculitic-mediated process driven by immunothrombosis rather than conventional thromboembolism. 

Rituximab was selected over cyclophosphamide for remission maintenance given its favorable safety profile. While both agents demonstrate comparable efficacy in MPO-ANCA-positive vasculitis, rituximab is associated with lower risks of malignancy, infertility, and bladder toxicity compared to cyclophosphamide. Current guidelines support the use of either agent in severe disease, with rituximab often preferred when renal function is preserved and no contraindications exist [1]. In this patient, preserved renal function and prior comorbidities supported rituximab use. Given her history of resolved hepatitis B infection, appropriate antiviral prophylaxis with entecavir 0.5 mg daily and monitoring were implemented due to the risk of viral reactivation with anti-CD20 therapy.

A retrospective multicenter study of 1,304 patients with systemic vasculitis reported digital necrosis in only 3.1% of cases, underscoring the rarity of this manifestation [3]. Prior case reports, particularly in PR3-ANCA-positive GPA, have demonstrated progression to gangrene requiring amputation despite immunosuppressive therapy [4,5]. Similarly, in this case, immunosuppression with glucocorticoids and maintenance with rituximab halted further progression but did not reverse established ischemia, ultimately necessitating amputation. This highlights that while early treatment may prevent additional vascular injury, irreversible tissue damage may persist once necrosis has developed. At follow-up, the patient has achieved serologic remission without recurrent ischemic events. This clinical course mirrors findings in the literature, in which immunosuppression may limit further vascular injury but does not reverse tissue necrosis once established.

This case supports the concept of immunothrombosis as a mechanism of vascular occlusion in autoimmune disease, including ANCA-associated vasculitis and rheumatoid vasculitis. Prior studies have demonstrated an increased risk of venous thromboembolism in patients with active vasculitis, particularly in the presence of renal involvement [10-12]. Emerging data also suggest an increased risk of arterial thrombotic events, including myocardial infarction and stroke [11]. Future studies are needed to better define the role of anticoagulation in thrombosis and arterial ischemia associated with ANCA vasculitis. Although the role of anticoagulation in AAV-associated thrombosis remains uncertain, it is frequently initiated despite the absence of a conventional thromboembolic source. Consistent with prior reports, anticoagulation alone was insufficient in this case [4,5]. Antithrombotic therapies, including low-dose aspirin or systemic anticoagulation, may be considered on an individualized basis according to thrombotic risk. However, immunosuppressive therapy is generally regarded as the primary treatment approach for thrombosis associated with AAV, as vascular occlusion is largely driven by inflammation-mediated endothelial injury rather than primary coagulation abnormalities [6-11]. The pathogenesis of thrombosis in AAV is thought to involve immunothrombosis driven by neutrophil activation, endothelial injury, and the formation of NETs, which promote tissue factor expression, platelet activation, and the amplification of the coagulation cascade [6-10]. As such, vascular occlusion may occur despite anticoagulation, as endothelial inflammation rather than primary coagulation pathway activation appears to be the dominant mechanism. This mechanism likely explains the progression of ischemia despite anticoagulation and the subsequent stabilization following immunosuppressive therapy in our patient.

This case also highlights the diagnostic complexity in patients with pre-existing autoimmune disease. The patient's history of seropositive rheumatoid arthritis initially raised concern for rheumatoid vasculitis, and the presence of nonbacterial thrombotic (marantic) vegetations further complicated the differential diagnosis. Additionally, a history of medication nonadherence likely contributed to the long-standing manifestations of uncontrolled rheumatoid disease, including suspected CTD-ILD, scleromalacia perforans, and chronically elevated rheumatoid factor. However, at the time of presentation, there were no clinical or serologic features suggestive of active rheumatoid vasculitis, including stroke, scleritis, worsening respiratory status from baseline, vasculitic ulcerations or nodules, or new joint erosions, thereby lowering the likelihood of this diagnosis.

In this context, the patient did not meet the 2022 American College of Rheumatology (ACR)/European Alliance of Associations for Rheumatology (EULAR) classification criteria for GPA [2]. Using these criteria, she would score MPO-ANCA positivity (−1 point), granuloma on biopsy (+2 points), and absence of pauci-immune glomerulonephritis (0 points), for a total of +1 point (threshold ≥5 points required). However, classification criteria are designed to identify homogeneous cohorts for clinical research and are not intended to serve as diagnostic criteria in individual patients. This distinction is particularly important in atypical or limited disease presentations. In clinical practice, diagnosis may still be established in patients with atypical or limited disease when supported by serologic, histopathologic, and clinical findings. Given the positive MPO-ANCA, histopathology consistent with granulomatous vasculitis, absence of alternative diagnoses, and immediate response to immunosuppression, a clinical diagnosis of MPO-ANCA-positive GPA was made. Notably, the presence of granulomatous inflammation on biopsy favored a diagnosis of GPA rather than MPA, which typically lacks granuloma formation.

Additionally, the absence of prior vasculitic episodes despite a long-standing history of rheumatoid arthritis, negative blood cultures excluding an infective etiology of the valvular vegetations, and cardiology assessment that the nonbacterial thrombotic (marantic) vegetations were small and unlikely to be clinically significant all supported a diagnosis of GPA. It remains uncertain whether the patient had subclinical or intermittent vasculitic activity previously, with this presentation representing an acute severe flare. Given the diagnostic overlap between rheumatoid vasculitis and GPA, an overlap syndrome cannot be entirely excluded.

Hepatitis B virus (HBV) DNA testing was not performed; however, the presence of anti-HB seroconversion and negative HBsAg was consistent with prior resolved infection and argued against active hepatitis B. Low protein C (37%) and antithrombin III (75%) levels were most consistent with acquired deficiencies due to inflammation-mediated consumption and cytokine-induced downregulation rather than hereditary thrombophilia. Despite these diagnostic complexities, management and prognostic considerations were appropriately guided by immunosuppressive therapy targeting systemic inflammation.

## Conclusions

Acute digital arterial ischemia leading to necrosis is a rare but severe manifestation of MPO-ANCA-positive GPA. Vasculitis should be considered in patients with autoimmune disease who present with unexplained or progressive digital ischemia, particularly when symptoms worsen despite anticoagulation. Early recognition and prompt immunosuppressive therapy may halt disease progression and prevent the involvement of additional digits, although established tissue necrosis may be irreversible. This case highlights the importance of distinguishing inflammatory vascular occlusion from conventional thromboembolic disease, as management strategies differ significantly. It also adds to the limited literature on atypical vascular manifestations of ANCA-associated vasculitis, particularly MPO-ANCA-positive GPA associated-phenotype, and highlights the role of immunothrombosis in disease pathogenesis.
